# Genetic alteration of human *MYH6* is mimicked by SARS-CoV-2 polyprotein: mapping viral variants of cardiac interest

**DOI:** 10.1038/s41420-022-00914-9

**Published:** 2022-03-21

**Authors:** Praveen Anand, Patrick J. Lenehan, Michiel Niesen, Unice Yoo, Dhruti Patwardhan, Marcelo Montorzi, A. J. Venkatakrishnan, Venky Soundararajan

**Affiliations:** 1 nference Labs, Bengaluru, Karnataka 560017 India; 2 nference, Cambridge, MA 02139 USA; 3 Southcoast Health, Fairhaven, MA 02719 USA

**Keywords:** RNA vaccines, Predictive markers

## Abstract

Acute cardiac injury has been observed in a subset of COVID-19 patients, but the molecular basis for this clinical phenotype is unknown. It has been hypothesized that molecular mimicry may play a role in triggering an autoimmune inflammatory reaction in some individuals after SARS-CoV-2 infection. Here we investigate if linear peptides contained in proteins that are primarily expressed in the heart also occur in the SARS-CoV-2 proteome. Specifically, we compared the library of 136,704 8-mer peptides from 144 human proteins (including splicing variants) to 9926 8-mers from all the viral proteins in the reference SARS-CoV-2 proteome. No 8-mers were exactly identical between the reference human proteome and the reference SARS-CoV-2 proteome. However, there were 45 8-mers that differed by only one amino acid when compared to the reference SARS-CoV-2 proteome. Interestingly, analysis of protein-coding mutations from 141,456 individuals showed that one of these 8-mers from the SARS-CoV-2 Replicase polyprotein 1a/1ab (KIALKGGK) is identical to an *MYH6* peptide encoded by the c.5410 C > A (Q1804K) genetic variation, which has been observed at low prevalence in Africans/African Americans (0.08%), East Asians (0.3%), South Asians (0.06%), and Latino/Admixed Americans (0.003%). Furthermore, analysis of 4.85 million SARS-CoV-2 genomes from over 200 countries shows that viral evolution has already resulted in 20 additional 8-mer peptides that are identical to human heart-enriched proteins encoded by reference sequences or genetic variants. Whether such mimicry contributes to cardiac inflammation during or after COVID-19 illness warrants further experimental evaluation. We suggest that SARS-CoV-2 variants harboring peptides identical to human cardiac proteins should be investigated as “viral variants of cardiac interest”.

## Introduction

Cardiac injury is a prevalent complication associated with COVID-19 [1]. In a study of 100 recently recovered COVID-19 patients, cardiovascular magnetic resonance imaging revealed cardiac involvement or ongoing myocardial inflammation in 78 and 60 patients, respectively [2]. In another study of 39 consecutive autopsies from patients who died of COVID-19, viral RNA was detectable in the heart of 24 (62%) patients. A large nationwide study from Israel reported that SARS-CoV-2 infection is associated with increased rates of myocarditis, arrhythmia, myocardial infarction, and pericarditis [3]. Myocarditis has also been reported in a small fraction of individuals after receiving an mRNA COVID-19 vaccine [4–6].

Despite these phenotypic associations, the mechanisms underlying myocardial inflammation in the setting of COVID-19 infection and vaccination remain unclear. A prevalent hypothesis, known as molecular mimicry, posits that T lymphocytes and/or antibodies that recognize SARS-CoV-2 antigens and mediate virus neutralization may also cross-react against host cardiac proteins and trigger an autoimmune response against cardiomyocytes [7]. This mechanism has also been suggested to contribute to other inflammatory conditions seen in the context of COVID-19 infection [8–10]. Indeed, autoimmune sequelae of other infectious diseases have been attributed to mimicry between host and microbial antigens [11–16].

Advances in next-generation sequencing technologies have facilitated the rapid development of large-scale multi-omic datasets and genomic epidemiology resources to better understand the COVID-19 pandemic. Bulk and single-cell RNA-sequencing datasets have elucidated the transcriptional signatures of most healthy human tissues and cell types [17–20]. Amino acid sequences of human proteins, including genetic variants and immunologic epitopes, are available in UniProt [21], gnomAD [22], and Immune Epitope Database (IEDB) [23]. The GISAID database currently hosts 4.85 million SARS-CoV-2 genomes from more than 200 countries[24]. The availability of such genome-scale data enables us to investigate the potential for molecular mimicry between SARS-CoV-2 and human cardiac proteins.

Here we present a systematic comparison of peptides from human cardiac proteins and SARS-CoV-2 proteins. We show that no 8-mer peptides are identical between the reference sequences of these two groups of proteins. However, when including human and viral genetic variants in this comparison, we found 21 8-mer peptides to be identical between human cardiac proteins and SARS-CoV-2 proteins. Among these, a human genetic variant of *MYH6* (c.5410 C > A; Q1804K) is identical to a peptide of the reference SARS-CoV-2 replicase polyprotein. Finally, we propose that the SARS-CoV-2 variants that have peptides identical to human cardiac proteins should be studied as potential “viral variants of cardiac interest”.

## Results

### Identification of genes that are overexpressed in cardiac tissue

**Fig. 1:**
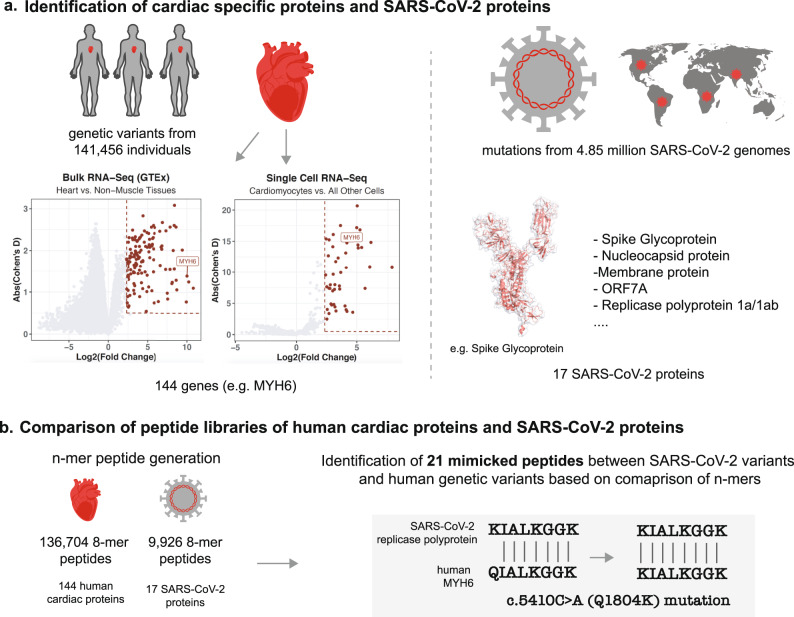
Identification of mimicked peptides between SARS-CoV-2 and human proteins. **a** Identification of cardiac-specific proteins based on analysis of bulk RNAseq and single-cell RNA-seq data and identification of SARS-CoV-2 proteins. **b** Comparison of peptide libraries of human cardiac proteins and SARS-CoV-2 proteins.

To identify heart-enriched proteins, we compared the expression of all human protein-coding genes in heart samples (*n* = 861) versus all non-striated muscle samples (*n* = 15,718) from the Genotype-Tissue Expression (GTEx) project. There were 137 genes expressed at least fivefold higher in the heart with a Cohen’s D value greater than or equal to 0.5 (Fig. 1 and Supplementary Table 1). Similarly, we compared the expression of all genes between cardiomyocytes (*n* = 8.9 K cells) and non-cardiomyocytes (*n* = ~2.5 million cells) across a database of 52 single-cell RNA-sequencing studies covering 62 tissues[18]. There were 46 genes overexpressed in cardiomyocytes based on the same criteria outlined above (Fig. 1 and Supplementary Table 1). Combining the lists of genes identified from bulk and single-cell RNA-sequencing analyses, we identified a total of 144 candidate cardiac proteins.

### *MYH6* variant is mimicked by an epitope of SARS-CoV-2 Replicase polyprotein 1a/1ab

We computed peptide 8-mers from the reference sequences of the 144 cardiac proteins (including isoforms) and all 17 proteins from the reference SARS-CoV-2 sequence. We then systematically compared the pairwise sequence identity (using Hamming Distance; see Methods) for the 136,704 cardiac protein 8-mers with the 9,926 8-mers derived from the SARS-CoV-2 proteins. No peptides were fully identical between these two groups. However, 45 8-mers were nearly identical, with only a single mismatched amino acid (Table 1). To determine whether human genetic variation results in any 8-mers, which exactly match the reference SARS-CoV-2 proteome, we then analyzed amino acid mutations from 141,456 individuals using the gnomAD database (including 83,623 mutations in the 144 cardiac proteins) [22].

**Table 1:** List of peptide pairs from SARS-CoV-2 proteins and human cardiac proteins that have a Hamming distance less than or equal to 1.

SARS-CoV-2 Proteins			Human cardiac-enriched proteins		
Protein name	Amino acid positions	Amino acid sequence	Protein name	Isoforms containing sequence	Amino acid sequence
Spike glycoprotein WT/2 P	491–498	PLQSYGFQ	KLHL41	1, 2	PLQSYFFQ
Spike glycoprotein WT/2 P	856–863	NGLTVLPP	FHOD3	1, 2, 3, 4	IGLTVLPP
Spike glycoprotein WT/2 P	857–864	GLTVLPPL	FHOD3	1, 2, 3, 4	GLTVLPPP
Spike glycoprotein WT/2 P	1087–1094	AHFPREGV	CMYA5	1	AHFPAEGV
Replicase polyprotein 1ab	4937–4944	KYAISAKN	TTN	1, 3, 2, 5, 12, 13, 7, 8, 4, 9, 10, 11	KYIISAKN
Replicase polyprotein 1ab	5604–5611	LQGPPGTG	MYLK3	1, 4	KQGPPGTG
Replicase polyprotein 1ab	5605–5612	QGPPGTGK	MYLK3	1, 4	QGPPGTGR
Replicase polyprotein 1ab	5813–5820	NRPQIGVV	CASQ2	1, 2	FRPQIGVV
Replicase polyprotein 1ab	5814–5821	RPQIGVVR	CASQ2	1, 2	RPQIGVVN
Replicase polyprotein 1ab	5955–5962	DTKFKTEG	TTN	1, 3, 2, 5, 12, 13, 7, 8, 4, 9, 10, 11	DTKFKTTG
Replicase polyprotein 1ab	5955–5963	DTKFKTEGL	TTN	1, 3, 2, 5, 12, 13, 7, 8, 4, 9, 10, 11	DTKFKTTGL
Replicase polyprotein 1ab	5956–5963	TKFKTEGL	TTN	1, 3, 2, 5, 12, 13, 7, 8, 4, 9, 10, 11	TKFKTTGL
Replicase polyprotein 1ab	6516–6523	KPVPEVKI	CMYA5	1	KPSPEVKI
Replicase polyprotein 1ab	6516–6523	KPVPEVKI	TTN	1, 2, 5, 12, 13, 7, 8, 4, 11	KPVPEEKI
Replicase polyprotein 1a/1ab	207–214	RAGKASCT	TTN	1, 2, 5, 12, 13, 7, 8, 4, 11	EAGKASCT
Replicase polyprotein 1a/1ab	208–215	AGKASCTL	TTN	1, 2, 5, 12, 13, 7, 8, 4, 11	AGKASCTT
Replicase polyprotein 1a/1ab	345–352	GTENLTKE	TNNI3K	1, 3, 4	GTESLTKE
Replicase polyprotein 1a/1ab	459–466	VNINIVGD	ENO3	1, 3, 2	VNIQIVGD
Replicase polyprotein 1a/1ab	492–499	KGLDYKAF	CASQ2	2	KKLDYKAF
Replicase polyprotein 1a/1ab	512–519	TKGKAKKG	MYH7	1	GKGKAKKG
Replicase polyprotein 1a/1ab	513–520	KGKAKKGA	MYH7	1	KGKAKKGS
Replicase polyprotein 1a/1ab	879–886	VIKTLQPV	LMOD3	1	VIKTLKPV
Replicase polyprotein 1a/1ab	963–970	GATSAALQ	ANKRD2	1, 2	GAQSAALQ
Replicase polyprotein 1a/1ab	1143–1150	VLLAPLLS	HJV	1, 2, 3	TLLAPLLS
Replicase polyprotein 1a/1ab	1144–1151	LLAPLLSA	HJV	1, 2, 3	LLAPLLSG
Replicase polyprotein 1a/1ab	1197–1204	KQVEQKIA	GOT1	1, 2	KKVEQKIA
Replicase polyprotein 1a/1ab	2246–2253	STAALGVL	SLC4A3	1, 2, 3	STAVLGVL
Replicase polyprotein 1a/1ab	2533–2540	KGSLPINV	TTN	1, 2, 5, 12, 13, 7, 8, 4, 11	KGSLPITV
Replicase polyprotein 1a/1ab	2550–2557	EESSAKSA	MYH7B	1	EESKAKSA
Replicase polyprotein 1a/1ab	2630–2637	LSTFISAA	TENM2	1, 2	LSTFFSAA
Replicase polyprotein 1a/1ab	2757–2764	KIALKGGK	MYH6	1	QIALKGGK
Replicase polyprotein 1a/1ab	2757–2764	KIALKGGK	MYH7	1	QIALKGGK
Replicase polyprotein 1a/1ab	2758–2765	IALKGGKI	MYH6	1	IALKGGKK
Replicase polyprotein 1a/1ab	2758–2765	IALKGGKI	MYH7	1	IALKGGKK
Replicase polyprotein 1a/1ab	3908–3915	FEKMVSLL	MYH6	1	EEKMVSLL
Replicase polyprotein 1a/1ab	3908–3915	FEKMVSLL	MYH7	1	EEKMVSLL
Replicase polyprotein 1a/1ab	3909–3916	EKMVSLLS	MYH6	1	EKMVSLLQ
Replicase polyprotein 1a/1ab	3909–3916	EKMVSLLS	MYH7	1	EKMVSLLQ
Replicase polyprotein 1a/1ab	4137–4144	VKLQNNEL	TBX20	1	VKLTNNEL
Putative ORF9c protein	47–54	AAVGELLL	ASB10	1, 2, 3	AAVVELLL
ORF7b protein	14–21	LAFLLFLV	TMEM182	2, 1, 3	LAGLLFLV
ORF7a protein	43–50	NSPFHPLA	FLNC	1, 2	NSPFHVLA
Nucleoprotein	192–199	NSSRNSTP	CMYA5	1	NSSRSSTP
Nucleoprotein	374–381	KKADETQA	MYPN	1	EKADETQA
Nucleoprotein	375–382	KADETQAL	MYPN	1	KADETQAR

Interestingly, one specific 8-mer from the SARS-CoV-2 Replicase polyprotein 1a/1ab (KIALKGGK) is identical to a mutant peptide encoded by the c.5410 C > A(Gln1804Lys) variation in human *MYH6* (**K**IALKGGK), which is a subunit of a cardiac motor protein. This genetic variation has been identified in Africans/African Americans (0.08% prevalence), East Asians (0.3% prevalence), South Asians (0.06% prevalence) and Latino/Admixed Americans (0.003% prevalence) [22]. Analysis of peptides from IEDB shows that the non-mutated 7-mer in this peptide (IALKGGK) also overlaps with a known B-cell epitope from SARS-CoV-2 (IALKGGKIVNNWLKQ, IEDB ID:1039277) [25]. Whether the mimicry between SARS-CoV-2 Replicase polyprotein 1a/1ab and wild-type or mutant *MYH6* contributes to cardiac inflammation in the setting of COVID-19 warrants further investigation.

### SARS-CoV-2 variants harbor epitopes that are identical to peptides of cardiac proteins

We next examined whether SARS-CoV-2 evolution has given rise to variants that harbor peptides identical to human cardiac proteins. To this end, we analyzed 4.85 million SARS-CoV-2 genomes from over 200 countries obtained from the GISAID database [24]. We identified 21 8-mer peptides from SARS-CoV-2 variants that are identical to cardiac proteins encoded by reference sequences or genetic variants (Tables 2 and 3). Of these 21 peptides, the one present in the highest number of viral sequences was STAVLGVL, which mapped to the NSP3 protein (part of Replicase polyprotein sequence) in 4501 (0.09%) SARS-CoV-2 genomes. This sequence is present in a transmembrane helix of all three described isoforms of the cardiac protein SLC4A3 (UniProtKB: P48751), an anion exchange protein that is associated with short QT syndrome (Tables 2 and 3) [21, 26]. Although no epitopes containing this full sequence were present in IEDB, a BLAST search at 90% similarity identified a similar epitope (HEAQAVLGVLL) that is reported to bind to both HLA-B*40:1 and HLA-B*58:01 [25, 27]. We additionally analyzed the temporal and geographical emergence of the five variant SARS-CoV-2 8-mers that are identical to cardiac protein 8-mers and that occur in over 100 reported SARS-CoV-2 genomes (Supplementary Fig. S1). Variants with exactly matching 8-mers have occurred sporadically in different geographic locations, but they have not persisted over time (Supplementary Fig. S1).

**Table 2:** List of identical cardiac peptides found in SARS-CoV-2 variants in GISAID.

No. of GISAID entries with identical match	SARS-CoV-2 gene	Mimicked peptide	Approx. start-end in SCOV2 protein	Cardiac protein	Cardiac protein start-end	Exact IEDB epitope match	Identical IEDB epitope seq (HLA/antigen infor) [≥90% identity]
4501	NSP3	STAVLGVL	1429–1436	SLC4A3	746–753	No	HEAQAVLGVLL (HLA-B*40:01); HEAQAVLGVLL (HLA-B*40:01)
1322	NSP2	GTESLTKE	166–173	TNNI3K	294–301	No	NA
580	N	NSSRSSTP	193–200	CMYA5	88–95	No	TINSSRSSQESY (B-cell epitopes, MHC ligands)
205	NS9c	AAVVELLL	48–55	ASB10	307–314	No	MVDPQLDGPQLAALA AVVELGSFDA ()
118	NSP2	KGKAKKGS	334–341	MYH7	635–642	Yes	AGADAPIEKGKGKAKKGSS (MHC ligand)
33	NSP3	VIKTLKPV	60–67	LMOD3	514–521	No	KVAIKTLKPGTMS (HLA-A*02:01);
							TKVAIKTLKPGTMSPE (HLA-A*02:01)
17	NSP3	KKVEQKIA	380–387	GOT1	55–62		GEKVEQKIEGKWVNEKKAQEDKLQ
							(MHC Class I, II, B-cell epitope)
14	NS7a	NSPFHVLA	44–51	FLNC	1727–1734	No	NA
11	N	EKADETQA	375–382	MYPN	89–96	No	ADETQALPQRQKKQQ (HLA Class II)
9	NSP3	TLLAPLLS	304–311	HJV	409–416		NFNQHEVLLAPLLS (B-cell epitope and MHC ligand)
6	NSP3	LSTFFSAA	1811–1818	TENM2	1036–1043	No	AGTLSTFFGVPLVLT (HLA class II MHC restriction)
6	NSP3	LLAPLLSG	327–334	HJV	410–417	No	ENFNQHEVLLAPLLS
							(B cell and MHC)
6	Spike	IGLTVLPP	854–861	FHOD3	971–978	No	GFIKQYGDCLGDIAA RDLICAQKFNGLTVL PPLLTDEMIAQYT (T cell, B cell and MHC ligands)
2	NSP13	QGPPGTGR	282–289	MYLK3	373–380	No	ILYGPPGTGK (HLA-A*03:01)
1	NSP15	KPVPEEKI	65–72	TTN	10277–10284	No	EAPLYVVDKPVPEESE (HLA-DRB1*04:01)
1	NSP3	KGSLPITV	1716–1722	TTN	5437–5444	No	SLPITVYYAV (T cell, B cell and MHC ligands)

The NSP proteins are cleaved products of the replicase polyprotein.

**Table 3:** List of mutated cardiac peptide n-mers from human genetic variants identical to SARS-CoV-2 variants.

Human cardiac gene	rsID	Mutation consequence	Mutated cardiac peptide	Wild-type cardiac peptide	No. of GISAID genomes (Pango lineage distribution in %)	SARS-CoV-2 gene	IEDB epitope exact match	IEDB epitope info (≥90 % seq identity)
FLNC	rs374848954	p.Val1732Leu	NSPFHLLA	NSPFHVLA	1661 (AY.4: 30.8% AY.44: 10.6%; B.1.1.7: 9.4%; AY.43: 6.85%;	NS7a	Yes	NSPFH (HLA-A*01:01)
LMOD3	rs370869958	p.Lys519Arg	VIKTLRPV	VIKTLKPV	85 (B.1.617.2: 24.7%; AY.43: 20.98%; AY.4: 8.64%; B.1.1: 6.17%; B.1.1.7: 6.17%; P.1: 6.17%;)	NSP3	No	NA
TMEM182	rs774398171	p.Gly215Val	LAVLLFLV	LAGLLFLV	21 AY.3: 57.14%; B.1.243: 14.28%; B.1.1.7: 9.52%; B.1: 4.76%)	NS7b	No	NA
MYLK3	rs771870674	p.Arg380Cys	QGPPGTGC	QGPPGTGR	1 (B.1)	NSP13	No	GPPGTGKSHFAIGLA (B cell, T cell, MHC ligand)

The NSP proteins are cleaved products of the replicase polyprotein.

## Discussion

Understanding the mechanistic basis of acute cardiac injury in COVID-19 patients is important to develop countermeasures. Viral infections have previously been proposed to trigger autoimmune reactions, and it has been hypothesized that molecular mimicry plays a role in mediating autoimmune reactions [7–9, 11–13, 28]. Here, a systematic analysis of gene expression from cardiac tissues, based on bulk RNA-seq data and single-cell RNA-seq data, combined with analysis of human genetic variation and SARS-CoV-2 genomes has led to the identification of candidate proteins and peptide regions therein that might be involved in immune cross-reactivity (Tables 2 and 3). These newly identified identical peptides expand the set of known shared peptides between the human proteome and SARS-CoV-2, such as the furin cleavage site “RRARSVAS” present in both human ENaC-ɑ and the Spike glycoprotein[10, 29, 30]. Further research is warranted to ascertain whether these mimicked peptides contribute to autoinflammatory pathology in the context of COVID-19 infection. Additionally, given the occurrence of myocarditis in some individuals shortly after receiving an mRNA COVID-19 vaccine, the potential for molecular mimicry between the antigen encoded by these vaccines (i.e., the pre-fusion stabilized Spike glycoprotein) and human cardiac proteins should be evaluated[4–6, 31, 32].

There are a few limitations to this study. First, the human proteins that we surveyed were shortlisted based on their overexpression in cardiac tissue. There could be mimicked proteins that are shared between cardiac tissues and other tissues that are not accounted for in the current analysis. Second, there are other mechanisms that could contribute to autoimmunity after a viral infection such as bystander activation, epitope spreading, and viral persistence [33]. Third, the presence of identical peptides in cardiac proteins and the SARS-CoV-2 proteome could occur due to chance. Comparing all SARS-CoV-2 8-mers to a set of brain enriched proteins and a set of skin enriched proteins shows a similar probability distribution of Hamming distance (Supplementary Fig. S2), suggesting that the observed similarity with SARS-CoV-2 peptides is not specific to human cardiac proteins. Fourth, it is possible that peptides with lower degrees of similarity could contribute to immunologic mimicry, as T cells can be highly cross-reactive against different major histocompatibility complex (MHC)-presented peptides [34–38].

Taken together, by studying the intersection of human genetic variation in cardiac proteins and SARS-CoV-2 evolution, we have identified candidates of molecular mimicry that have the potential to contribute to cardiac inflammation in the context of COVID-19. It will be important to perform follow-up functional studies evaluating the potential of SARS-CoV-2 reactive T cells and antibodies (e.g., from active or recovering COVID-19 patients) to cross-react with these peptides. Thus, we propose that SARS-CoV-2 variants harboring peptides identical to host heart-enriched proteins should be studied as “viral variants of cardiac interest”. We highlight that a similar strategy can be applied to identify and categorize plausible mimicry candidates from any human tissues that are targeted by other autoimmune responses in COVID-19 patients.

## Methods

### Identification of proteins enriched in cardiac tissue

Bulk RNA-sequencing (RNA-seq) data was accessed from the Genotype-Tissue Expression (GTEx) project V8 [17]. For each sample, FASTQ files were processed using Salmon (in mapping-based mode) to quantify gene expression in transcripts per million (TPM). Specifically, the expression of each transcript isoform was first determined by passing FASTQ files to Salmon *quant* with the following parameters passed: validateMappings, rangeFactorizationBins 4, gcBias, biasSpeedSamp 10. All isoforms are then summed via a transcript-to-gene map, generating a gene-level expression value. GRCh38 was used as the reference, including cDNA and non-coding RNA.

For single-cell RNA-seq studies, processed count matrices were accessed from Gene Expression Omnibus or other publicly available data repositories. There were two datasets analyzing heart tissues which captured cardiomyocytes, our main cell type of interest for this report [20, 39]. Other datasets captured a wide variety of immune, stromal, and parenchymal cell types from tissues including the respiratory tract, gastrointestinal tract, genitourinary tract, hepatobiliary system, skeletal muscle, brain, skin, eyes, and endocrine organs. Each dataset was processed using Scrublet and Seurat v3.0 as described previously [18, 40–42]. Cell type annotations were obtained from associated metadata files if available; otherwise, annotation was performed manually, guided by the cell types reported in the associated publication.

To identify genes that are overexpressed in cardiac tissue, we calculated fold change and Cohen’s D values between defined sample cohorts. For bulk RNA-seq data, Cohort A was defined as all GTEx heart samples (*n* = 861), and Cohort B was defined as all remaining GTEx samples except for those derived from skeletal muscle (*n* = 15,718). For single-cell RNA-seq data, Cohort A was defined as all cells annotated as cardiomyocytes (*n* ~8900 cells), and Cohort B was defined as all other cells from all processed studies (*n* ~2.5 million cells). Fold change and Cohen’s D were calculated as follows:

$${\mathbf{Fold}}\,{\mathbf{Change}} = \frac{{{\mathrm{TPM}}_{{\mathrm{Cohort}}\,{\mathrm{A}}} + 1}}{{{\mathrm{TPM}}_{{\mathrm{Cohort}}\,{\mathrm{B}}} + 1}}$$

**Cohen’s D** = $\frac{{{\mathrm{TPM}}_{{\mathrm{Cohort}}\,{\mathrm{A}}} - {\mathrm{TPM}}_{{\mathrm{Cohort}}\,{\mathrm{B}}}}}{{{\mathrm{SD}}_{{\mathrm{pooled}}}}}$, where the pooled standard deviation SD_pooled_ is defined as: **SD**_**pooled**_ = $\sqrt {\frac{{(N_{{\mathrm{Cohort}}\,A} - 1) \times {\mathrm{SD}}_{Cohort\,A}^2 + (N_{{\mathrm{Cohort}}\,B} - 1) \times {\mathrm{SD}}_{{\mathrm{Cohort}}\,B}^2}}{{(N_{{\mathrm{Cohort}}\,A} + N_{{\mathrm{Cohort}}\,B} - 2)}}}$, where *N*_Cohort A_ and *N*_Cohort B_ are the number of samples in Cohorts A and B, respectively, and SD_Cohort A_ and SD_Cohort B_ are the standard deviation of TPM values for the given gene in Cohorts A and B, respectively.

Genes with fold change ≥5 and Cohen’s D ≥ 0.5 from either the bulk or single-cell RNA-seq analysis were considered to be enriched in cardiac tissue. In the volcano plots used to visualize these analyses, we filtered to genes with a TPM or CP10K value ≥1 in either Cohort A or Cohort B, and genes meeting the criteria for overexpression in heart or cardiomyocytes are colored in red.

For a control analysis, we also identified genes overexpressed in the brain or skin by bulk RNA-sequencing from the GTEx project. We used the same approach as described above, except that Cohort A was defined as either all brain samples (*n* = 2351) or all skin samples (*n* = 1305), and Cohort B was defined as all other samples.

### Comparison of 8-mers from reference sequences of cardiac proteins and SARS-CoV-2 proteins

The translated proteome from reference to the SARS-CoV-2 genome (NC_045512.2) was downloaded from UniProt (https://covid-19.uniprot.org/) [21, 43]. A sliding window approach was used to enumerate all 8-mers from the 17 proteins in this viral proteome. Similarly, we used a sliding window approach to generate all 8-mers from the reference amino acid sequences of the previously defined 144 cardiac proteins, including the canonical isoforms and all described isoforms indicated in UniProt. We then performed a pairwise comparison of all 8-mers in these two groups by calculating the Hamming distance using the *stringdist* function from the stringdist package (version 0.9.8) in R (version 4.0.3). In a control analysis, we used the same approach to calculate the Hamming distance between all SARS-CoV-2 8-mers and the control sets of 369 human proteins enriched in the brain or 198 human proteins enriched in the skin (described above).

### Assessing the impact of human and SARS-CoV-2 variants on cardiac peptide matches

To assess the impact of human genetic variation on potential molecular mimicry, we retrieved all missense variants from the gnomAD database for the previously identified cardiac proteins that had at least one 8-mer similar to a peptide in the SARS-CoV-2 reference proteome (Hamming distance = 1) [22]. We used the gnomad-api (https://gnomad.broadinstitute.org/api) to fetch the variant calls from the gnomad_r2_1 version from the Human GRCh37 genome assembly. The variants in this gnomad version (GRCh37/hg19) are derived from 125,748 exome sequences and 15,708 whole-genome sequences from unrelated individuals sequenced as part of various disease-specific and population genetic studies. For any variants that alter the amino acid sequence of a potentially mimicked peptide, we determined whether the mutation resulted in an exact match (Hamming distance = 0) to the corresponding 8-mer from the SARS-CoV-2 reference proteome.

To assess the impact of viral evolution on potential molecular mimicry, we queried the cardiac 8-mers with Hamming distance of 1 (including any alterations of these 8-mers arising from human genetic variation as described above) against all protein variants encoded in 4,854,709 SARS-CoV-2 genomes deposited in the GISAID database (last accessed 11/5/2021) [24]. Here, we determined whether any mutations in viral genomes (relative to the reference sequence) resulted in 8-mers which exactly match one or more cardiac peptides.

### Evaluation of mimicked peptides for inclusion in immune epitopes

For any 8-mers which showed an exact match between a cardiac peptide (reference or variant sequences) and a SARS-CoV-2 peptide (reference or variant sequences), we queried the 8-mer using the Immune Epitope Database (IEDB; www.iedb.org) and Analysis Resource [23]. We searched for any linear peptide epitope with a Blast similarity of at least 90% from any human host that had positive experimental evidence in any assay (T cell, B cell, or MHC Ligand). No MHC class restrictions or disease filters were applied.

## Supplementary information

Supplementary Material

## Data Availability

The expression profiling analyses for the cardiac proteins were carried out using the count matrix derived from Genotype-Tissue Expression (GTEx) project V8 dataset (https://gtexportal.org/home/datasets). The data for single-cell datasets from heart tissue were reanalyzed from two different publicly available studies with the following GEO accession IDs: GSE134355 [20], GSE109819 [39], and GSE121893 [39]. The human variants were obtained from the gnomad 2.1.1 version that is publicly available for download at http://gnomad.broadinsitute.org. The antigenicity potential for the peptide matches were evaluated using the publicly accessible IEDB database (www.iedb.org).
