# Supplementary material for: Genetic alteration of human MYH6 is mimicked by SARS-CoV-2 polyprotein: mapping viral variants of cardiac interest
Source: Cell Death Discov. 2022 Mar 21;8:124. doi: 10.1038/s41420-022-00914-9 (PMC8935120; doi:10.1038/s41420-022-00914-9)
Supplement: Supplementary file 1 — Supplementary Material [file 41420_2022_914_MOESM1_ESM.docx]

**Supplementary Material**


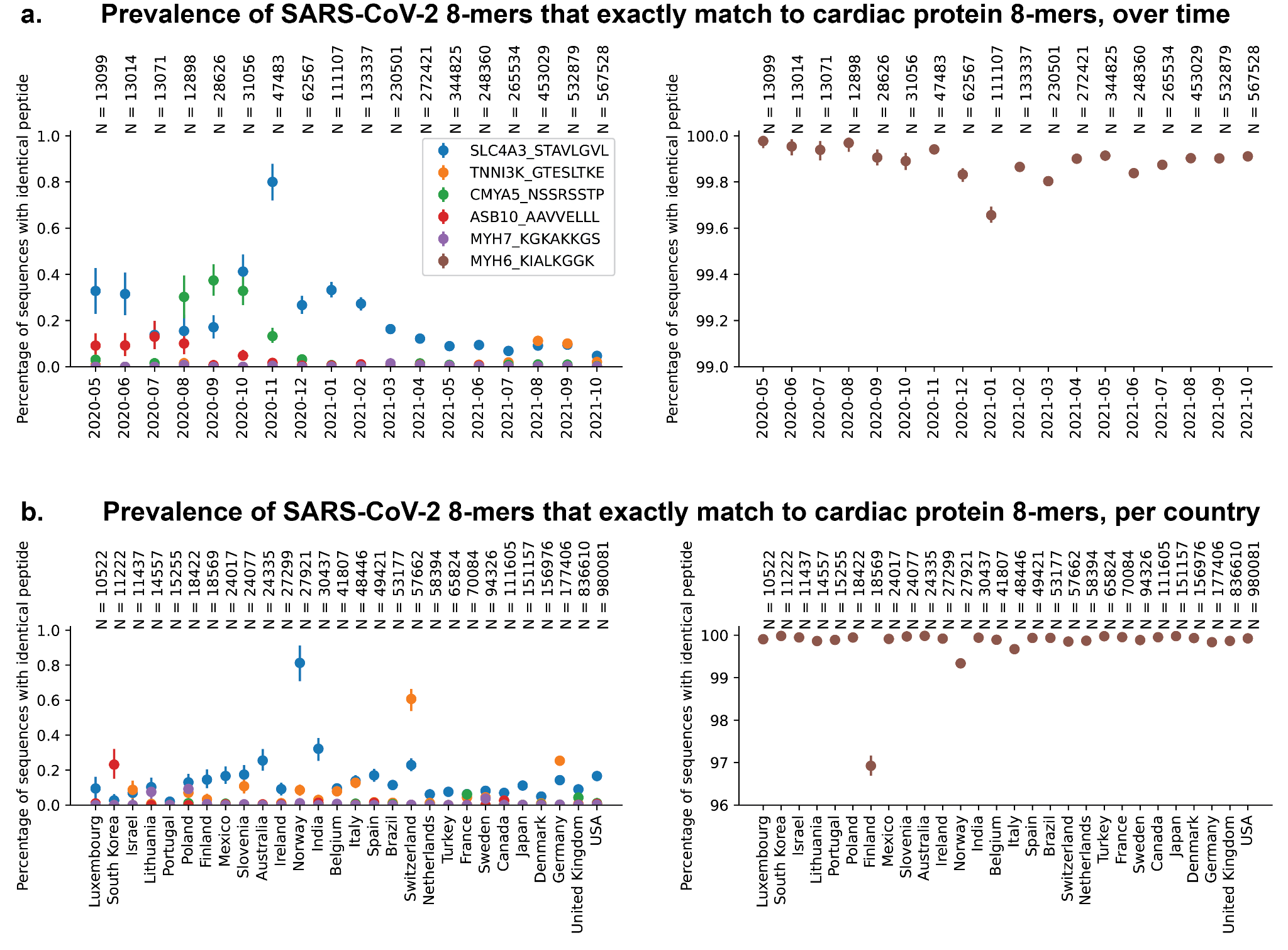


**Supplementary Figure S1: Prevalence of SARS-CoV-2 variants with exact 8-mer matches to human cardiac proteins.** Prevalence is reported as the percentage of SARS-CoV-2 sequences with exact 8-mer matches during 1-month time intervals (**a**) or across all sequences reported by a country (**b**). Prevalence of the wild-type SARS-CoV-2 8-mer that exactly matches with the MYH6 peptide, described in the main text, is shown separately on the right (brown). Data is only shown for months and countries in which more than 10,000 SARS-CoV-2 genomes were reported to GISAID.

**
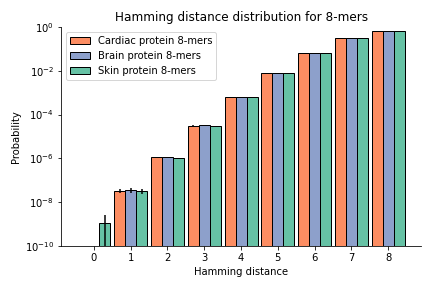
**

**Supplementary Figure S2. Observed probability distribution for the Hamming distance between all 8-mers in the SARS-CoV-2 proteome and all 8-mers in reference sets of human proteins.** Shown is the distribution of Hamming distance between all SARS-CoV-2 linear 8-mer peptides (n=9,926 peptides) and all linear 8-mer peptides from canonical isoforms of human cardiac proteins (orange, n=129,415 peptides), brain proteins (blue, n=241,046 peptides), and skin proteins (green, n=88,628 peptides). Error bars represent 95% confidence intervals.

**Supplementary Table S1. List of human cardiac proteins identified based on Bulk RNA-seq and Single cell RNA-seq. Rows with human genes from Tables 2 and 3 are highlighted.**

| **Gene** | **Bulk RNAseq Cohen'sD** | **Bulk RNAseq Fold Change** | **Single cell RNAseq Cohen'sD** | **Single cell RNAseq Fold Change** |
| --- | --- | --- | --- | --- |
| *NPPA* | 1.1 | 1796.96 | 10.81 | 228.63 |
| *MYL7* | 1.25 | 1206.31 | 13.98 | 41.82 |
| *MYH6* | 1.39 | 1035.51 | 14.39 | 33.95 |
| *NPPB* | 0.77 | 978.15 | 7.39 | 3.08 |
| *TNNI3* | 2.01 | 688.88 | 13.9 | 34.44 |
| *MYL2* | 1.06 | 426.75 | 14.8 | 70.24 |
| *MYH7* | 1.29 | 403.97 | 20.66 | 32.61 |
| *MYBPC3* | 2.56 | 398.67 | 14.13 | 7.9 |
| *TNNT2* | 3.08 | 349.01 | 15.97 | 40.17 |
| *CSRP3* | 1.56 | 342.89 | 17.53 | 12.22 |
| *MB* | 2.6 | 339.57 | 10.75 | 16.91 |
| *NMRK2* | 2 | 312.46 | 11.29 | 3.98 |
| *MYL3* | 1.24 | 287.28 | 12.33 | 10.69 |
| *MYL4* | 1.21 | 278.12 | 4.43 | 8.92 |
| *COX6A2* | 2.56 | 238.14 | 13.13 | 16.49 |
| *ANKRD1* | 1.55 | 236.39 | 16.81 | 38.11 |
| *BMP10* | 0.84 | 192.77 | 0.02 | 1 |
| *XIRP1* | 0.98 | 186.09 | 11.67 | 3.3 |
| *TCAP* | 2.47 | 165.66 | 11.75 | 9.32 |
| *LMOD2* | 1.55 | 161.06 | 15.38 | 5.41 |
| *CKM* | 2.67 | 121.39 | 14.76 | 17.56 |
| *TNNC1* | 2.11 | 116.52 | 9.95 | 12.41 |
| *ACTC1* | 2.03 | 114.83 | 17.17 | 31.65 |
| *TECRL* | 2.08 | 108.63 | 15.24 | 6.41 |
| *NRAP* | 1.64 | 100.1 | 9.86 | 2.67 |
| *MYOZ2* | 2.46 | 96.37 | 16.1 | 8.11 |
| *ACTA1* | 0.78 | 85.4 | 7.79 | 11.48 |
| *NKX2-5* | 2.55 | 60.66 | 2.68 | 1.24 |
| *SMPX* | 2.41 | 58.97 | 11.02 | 3.39 |
| *ANKRD2* | 0.74 | 56.65 | 3.14 | 1.48 |
| *MYOM2* | 2.04 | 54.35 | 3.28 | 2.9 |
| *SYNPO2L* | 1.78 | 54.28 | 7.88 | 3.21 |
| *FABP3* | 2.6 | 53.66 | 4.9 | 7.11 |
| *DHRS7C* | 1.48 | 53.13 | 3.91 | 1.26 |
| *HRC* | 2.31 | 52.1 | 9.54 | 5.25 |
| *ACTN2* | 2.29 | 49.24 | 7.42 | 6.34 |
| *CKMT2* | 2.15 | 48.13 | 5.57 | 3.19 |
| *TRIM63* | 2.11 | 46.3 | 3.35 | 1.75 |
| *SLN* | 1.03 | 45.97 | 4.25 | 3.84 |
| *HSPB3* | 2.56 | 44.41 | 6.57 | 2.16 |
| *FBXO40* | 2.08 | 38.61 | 8.3 | 2.2 |
| *APOBEC2* | 1.83 | 38.37 | 2.02 | 1.33 |
| *SRL* | 2.27 | 37.54 | 5.02 | 1.85 |
| *MYOM3* | 2.14 | 36.29 | 3.86 | 1.64 |
| *UNC45B* | 1.97 | 35.35 | 4.96 | 1.59 |
| *TTN* | 2.41 | 34.78 | 10.85 | 65.81 |
| *SBK2* | 1.12 | 34.73 | 0.08 | 1 |
| *MYLK3* | 2.08 | 30.66 | 3.38 | 1.59 |
| *RPL3L* | 1.4 | 30.36 | 3.51 | 1.44 |
| *TRIM54* | 2.43 | 30.03 | 1.55 | 1.25 |
| *MYBPHL* | 1.01 | 29.58 | 3.8 | 1.64 |
| *XIRP2* | 0.54 | 28.03 | 12.68 | 6.12 |
| *CMYA5* | 2.1 | 26.54 | 7.28 | 11.68 |
| *SMYD1* | 1.48 | 25.33 | 6.66 | 1.58 |
| *FITM1* | 2.47 | 23.91 | 4.26 | 1.96 |
| *SCN5A* | 2.2 | 23.56 | 2.62 | 1.26 |
| *TRDN* | 2.83 | 21.15 | 7 | 4.6 |
| *LMOD3* | 1.74 | 20.88 | 2.32 | 1.78 |
| *MYPN* | 1.76 | 20.84 | 3.81 | 1.39 |
| *TRIM55* | 1.08 | 20.55 | 2.44 | 1.4 |
| *LDB3* | 2.37 | 19.06 | 7.97 | 6.87 |
| *SMCO1* | 1.45 | 18.71 | 4.26 | 1.25 |
| *KLHL31* | 1.73 | 18.44 | 5.15 | 1.73 |
| *LRRC14B* | 1.22 | 18.39 | 0.07 | 1 |
| *PXDNL* | 2.01 | 17.72 | 0.72 | 1.13 |
| *POPDC2* | 2.32 | 16.72 | 3.57 | 1.89 |
| *PPP1R3A* | 1.62 | 16.72 | 5.73 | 1.46 |
| *ABRA* | 0.89 | 15.75 | 5.75 | 1.63 |
| *MYO18B* | 1.96 | 15.7 | 3.25 | 1.38 |
| *HHATL* | 2.3 | 15.55 | 0.82 | 1.2 |
| *LRRC10* | 1.52 | 15.46 | 3.59 | 1.13 |
| *MYH7B* | 2.1 | 15.44 | 1.42 | 1.29 |
| *MYOM1* | 1.84 | 15.35 | 7.65 | 5.61 |
| *SGCG* | 2.28 | 15.18 | 2.37 | 1.45 |
| *ENO3* | 1.84 | 14.26 | 4.15 | 2.92 |
| *RYR2* | 1.81 | 14.09 | 4.02 | 5.77 |
| *TBX20* | 1.44 | 13.73 | 0.06 | 1 |
| *HSPB7* | 1.88 | 13.18 | 9.66 | 8.07 |
| *ADPRHL1* | 2.24 | 13.08 | 1.81 | 1.49 |
| *ALPK2* | 2.22 | 12.49 | 2.64 | 1.44 |
| *ASB11* | 1.67 | 12.1 | 0.22 | 1.02 |
| *CASQ2* | 1.93 | 11.98 | 7.04 | 3.65 |
| *TMEM182* | 1.95 | 11.83 | 1.64 | 1.45 |
| *PERM1* | 1.79 | 11.75 | 0.77 | 1.06 |
| *ASB15* | 1.63 | 11.34 | 1.24 | 1.18 |
| *TXLNB* | 1.7 | 11.25 | 2.56 | 1.74 |
| *MYZAP* | 1.97 | 11.15 | 3.56 | 2.28 |
| *ITGB1BP2* | 2.1 | 11.09 | 2.37 | 1.25 |
| *RD3L* | 1.99 | 10.81 | 1.21 | 1.1 |
| *LRRC39* | 1.92 | 10.76 | 1.1 | 1.23 |
| *SLC25A4* | 2.33 | 10.73 | 3.77 | 9.45 |
| *NEBL* | 2.26 | 10.45 | 3.36 | 6.36 |
| *FAM155B* | 1.6 | 10.34 | 1.02 | 1.11 |
| *HJV* | 1.58 | 10.24 | 1.84 | 1.19 |
| *KLHL41* | 1.15 | 10.21 | 1.67 | 1.27 |
| *ASB10* | 1.9 | 9.93 | 1.16 | 1.05 |
| *TBX5* | 1.46 | 9.89 | 3.04 | 1.45 |
| *METTL7B* | 0.96 | 9.85 | 0.15 | 1.02 |
| *RBM24* | 1.85 | 9.81 | 2.26 | 1.57 |
| *PKP2* | 1.65 | 9.56 | 1.59 | 1.89 |
| *KLHL38* | 1.2 | 9.49 | 0.06 | 1 |
| *SBK3* | 1.04 | 9.31 | 0.26 | 1.01 |
| *CORIN* | 1.46 | 9.19 | 1.59 | 1.26 |
| *CAVIN4* | 1.48 | 9.16 | 2.79 | 1.27 |
| *CAV3* | 2.26 | 8.63 | 0.9 | 1.07 |
| *COX7A1* | 2.49 | 8.45 | 7.43 | 50.55 |
| *SLC4A3* | 2.3 | 8.27 | 2.78 | 1.82 |
| *GATA4* | 1.94 | 7.67 | 0.6 | 1.14 |
| *PEBP4* | 1.85 | 7.67 | 0.7 | 1.25 |
| *MLIP* | 1.43 | 7.14 | 2.02 | 1.65 |
| *DOK7* | 1.53 | 7.13 | 0.23 | 1.03 |
| *RBM20* | 2.03 | 6.94 | 2 | 1.48 |
| *EEF1A2* | 1.87 | 6.78 | 1.28 | 1.35 |
| *CHRNE* | 0.92 | 6.65 | 0.03 | 0.99 |
| *ATP2A2* | 1.73 | 6.64 | 2.08 | 4.52 |
| *PLA2G5* | 1.82 | 6.58 | 1.04 | 1.54 |
| *PLPP7* | 2.31 | 6.2 | -0.05 | 0.99 |
| *FSD2* | 1.74 | 6.11 | 1.56 | 1.23 |
| *FLNC* | 0.67 | 6.11 | 7.38 | 6.69 |
| *CRYAB* | 1.3 | 5.93 | 2.47 | 5.66 |
| *S100A1* | 2.2 | 5.86 | 0.1 | 0.8 |
| *TENM2* | 1.36 | 5.86 | 0.4 | 1.08 |
| *TNNI1* | 0.64 | 5.85 | 1.55 | 1.27 |
| *PLN* | 1.4 | 5.77 | 7.23 | 8.69 |
| *CDH2* | 1.68 | 5.73 | 1.74 | 2.01 |
| *FHL2* | 1.04 | 5.7 | 0.48 | 1.35 |
| *CYP2J2* | 1.6 | 5.67 | 0.52 | 1.17 |
| *FGF12* | 1.65 | 5.5 | 1.48 | 1.91 |
| *CCDC141* | 1.6 | 5.48 | 1.07 | 1.36 |
| *TNNI3K* | 1.71 | 5.47 | 0.31 | 1.05 |
| *SLC5A1* | 0.89 | 5.47 | 0.88 | 1.24 |
| *TNNT1* | 0.74 | 5.31 | 0.63 | 1.4 |
| *FHOD3* | 1.52 | 5.29 | 0.23 | 1.05 |
| *CLIC5* | 1.62 | 5.1 | 1.58 | 1.74 |
| *TPM1* | 1.78 | 5.06 | 3.71 | 17.66 |
| *LRRC2* | 1.07 | 5.05 | 1.53 | 1.51 |
| *GOT1* | 1.8 | 5.03 | 1.21 | 1.88 |
| *DES* | 1.2 | 3.88 | 7.99 | 8.88 |
| *NEXN* | 1.05 | 2.92 | 4.73 | 7.42 |
| *MTRNR2L8* | 0.27 | 1.32 | 3.56 | 24.52 |
| *MTRNR2L1* | 0.31 | 1.2 | 3.98 | 16.82 |
| *MTRNR2L10* | 0.45 | 1.12 | 4.58 | 15.78 |
| *MTRNR2L3* | -0.08 | 1 | 6.99 | 7.85 |
| *MSRB3* | -0.35 | 0.65 | 4.82 | 8.38 |
